# Rumor detection on social media using hierarchically aggregated feature via graph neural networks

**DOI:** 10.1007/s10489-022-03592-3

**Published:** 2022-05-21

**Authors:** Shouzhi Xu, Xiaodi Liu, Kai Ma, Fangmin Dong, Basheer Riskhan, Shunzhi Xiang, Changsong Bing

**Affiliations:** College of Computer and Information Technology, China Three Gorges University, Yichang, 443002 China

**Keywords:** Rumor detection, Graph neural networks, Hierarchical aggregation, Rumor propagation

## Abstract

In the era of the Internet and big data, online social media platforms have been developing rapidly, which accelerate rumors circulation. Rumor detection on social media is a worldwide challenging task due to rumor’s feature of high speed, fragmental information and extensive range. Most existing approaches identify rumors based on single-layered hybrid features like word features, sentiment features and user characteristics, or multimodal features like the combination of text features and image features. Some researchers adopted the hierarchical structure, but they neither used rumor propagation nor made full use of its retweet posts. In this paper, we propose a novel model for rumor detection based on Graph Neural Networks (GNN), named *Hierarchically Aggregated Graph Neural Networks* (HAGNN). This task focuses on capturing different granularities of high-level representations of text content and fusing the rumor propagation structure. It applies a Graph Convolutional Network (GCN) with a graph of rumor propagation to learn the text-granularity representations with the spreading of events. A GNN model with a document graph is employed to update aggregated features of both word and text granularity, it helps to form final representations of events to detect rumors. Experiments on two real-world datasets demonstrate the superiority of the proposed method over the baseline methods. Our model achieves the accuracy of 95.7*%* and 88.2*%* on the Weibo dataset Ma et al. 2017 and the CED dataset Song et al. IEEE Trans Knowl Data Eng 33(8):3035–3047, 2019respectively.

## Introduction

Social media platforms have become an indispensable part of our daily life in the age of big data, which increase people’s ability to obtain and exchange information significantly. Users can post, forward and comment on any real-time information through various platforms. Therefore, microblog platforms like Sina Weibo, Twitter usually have higher flexibility and stronger interactivity, and information can even be completely diffused. The explosive growth of data usually leads to fake news and rumors. Since the lack of monitoring mechanisms, harmful information can easily flourish. A report issued by the Weibo official account shows that the Weibo platform handled 76,107 false information in 2020. Rumors on social media have become a serious concern in recent years, especially when a pandemic like Coronavirus Disease-19 (the COVID-19) outbreaks. Peace and order of the society may be affected by diverse misinformation.

Human beings are taking great efforts to fight against rumors. However, rumor detection is a challenging task due to the following aspects: 1) The confusing character of rumors. Identifying rumors from massive information has become harder and harder, since rumors are usually fabricated almost like the real news. Sometimes rumors can even mix the spurious with the genuine. Unless the events are clarified in time by the people or institutes concerned, the rumors will spread further. 2) The speed of rumor diffusion. The rapid growth of the information can lead to rumor spreading with incredibly high pace. Fake news always travels faster than normal news because it is good at creating panic and anxiety. 3) Rumors cover a wide variety, from entertainment to political news. There is a large number of working on rumor detection. Most of the conventional methods tend to use classification algorithms with manually extracted features, such as Support Vector Machine (SVM) [3], Random Forest [4] and Decision Trees [5]. Recent research has employed deep learning methods to explore high-level representations of rumors from text contents [6], spreading path [7], user information [8], sentiment tendency [9] and other features. Many methods capable to learn different types of features are proposed in literatures, such as the Recursive Neural Networks (RvNN) [10], Long Short Term Memory (LSTM), Gated Recurrent Unit (GRU) [1] and Convolutional Neural Networks (CNN) [11, 12]. There are still some challenges. Though plenty of research has been done on different perspectives, the propagation mechanism of rumors has not been studied adequately. It is still a problem to concretize the propagation patterns in terms of monitoring.

A CNN-based graph-structured semi-supervised approach named Graph Convolutional Networks (GCN) [13] was proposed in 2016. It can efficiently extract spatial features in the topological graph like rumor propagation graph. The Gated Graph Neural Networks (GGNN) [14] on textual documents can not only capture the contextual word relationships in documents but also implement the inductive learning of new words. Thus, we introduce GCN and GGNN in our rumor detecting model. This paper proposed a novel dual-grained feature aggregation graph neural network (HAGNN), which operates on GCN and GNN. The proposed method obtains the text features via GCN and acquires word-text aggregated features via GGNN. The main points of this work are as follows:

- Both GCN and GNN are adopted to detect rumors on different hierarchies, in which event graphs are constructed at the text level while text graphs are at the word level.
- The proposed HAGNN model uses both word-level representations and text-level updated vectors. Besides, the text-granularity features are generated through rumor propagation.
- To make comprehensive use of both source posts and retweet posts, the updated text features of the rumor are concatenated with the word features of the source post at the graph neural network module.

The rest of this paper is organized as follows. Section 2 presents the related work of rumor detection. Section 3 is the statement of variables and data structures. In Section 4, the proposed model and its modules are elaborated. Section 5 presents the experiments and analyzes the results. Section 6 is the conclusion.

## Related work

Automatic detection of rumors aims to identify rumors using series of approaches through plentiful information like text contents, comments and forwarding patterns on social media. Rumor detection methods in recent years can be grouped into three main categories: 1) conventional methods, 2) classical deep learning methods and 3) graph neural network methods.

### Conventional methods

Some researches focus on traditional handworked features and classification methods. Al-Ghadir et al. [15] proposed a novel enhanced method for identifying writer’s stance of a tweet. They selected the fusion of term frequency-inverse document frequency (tf-idf) scores and the sentiment information for generating feature vectors. SVM, K-NN and its variants were employed to evaluate the method. Chu et al. [16] used Predicting The Security Threats of Internet Rumors (PSTIR) and Spread of False Information Based on Sociological (SFIBS) to linearly analyzed the proportion of trustworthy Facebook fans that post regularly in early and future popularity. Askarizadeh et al. [17] proposed a soft rumor control model where people can refer to their trusted friends or ask the reputable authorities about the rumor to avoid rumor spreading. Nicolas et al. [18] studied the extend to which emotions explain the diffusion of online rumors. They analyzed a sample of online rumors and its cascades. Then they used a generalized linear regression model to estimate how emotions are associated with the spread of online rumors in terms of cascade size, cascade lifetime and structural virality.

Both the confidence model and credibility network can be used in rumor detection. Douven et al. [19] introduced typed agents into the bounded confidence model to study misinformation and disinformation campaigns. The agents can be irresponsible in different ways, but they are reluctant to try and obtain information from the world directly. They further added a mechanism of confidence dynamics to the model. The mechanism allows agents to adapt the threshold for counting others as being their neighbors. Srinivasan et al. [20] focused on a collective rumor containment approach to control or eradicate rumors. They proposed an anti-rumor information spreading approach to contain rumors collectively by following a bio-inspired immunization method named social immunity. It continuously updates the trustworthiness of individuals in the network on every communication among the participants. Jin et al. [21] used conflicting information on social media and built credibility propagation network to verify the news.

Both time features and similarities of propagation are also considered in the task. Ai et al. [22] improves the traditional scale-free network and proposed a topology model based on the network theory and the actual characteristics of sharing social networks. In addition, they proposed the credulous spider rational taciturn rumor propagation model, which solved the problem of overspread in traditional rumor propagation model. Aiming at the rumor source detection task, Fan et al. [23] presented a Belief-Propagation-based (BP) algorithm to compute the joint likelihood function of the source location and the spreading time for the general continuous-time Susceptible-Infected epidemic model on trees. Besides, they proposed a “Gamma Generated Tree” heuristic to convert an original graph to a tree, whose edges have heterogeneous infection rates.

### Classical deep learning methods

Several classic methods based on deep learning were proposed in recent years. Ma et al. contributed a series of work on rumor detection with neural network technology in [1, 10, 24, 25]. Using Recursive Neural Networks (RvNN), they proposed top-down and bottom-up tree-structured neural networks that relate text content to propagation clues [10]. Ma et al. [1] discovered the continuity of the text stream, and applied Recurrent Neural Networks (RNN) to capture the dynamic time information of rumor forwarding. They proposed a model based on RNN to learn the semantic features of tweet context over time. RNN is also utilized in multi-task learning. Ma et al. [24] also argue that rumor detection and stance classification should be treated as an entirety. Thus they proposed a joint framework that unifies the two pertinent tasks. It helps to learn rumor representations. Besides, inspired by Generative Adversarial Networks (GAN), Ma et al. [25] presented a GAN-style approach, in which a generator is designed to produce conflicting voices in order to learn stronger rumor indicative representations.

Spreading patterns and social network relations are considered in rumor detecting models based on neural networks. Alkhodair et al. [26] studied the problem of detecting breaking news rumors. They proposed an approach that jointly learns word embeddings and trains a recurrent neural network with two different objectives to automatically identify rumors. Zhang et al. [27] proposed a lightweight propagation path aggregating (PPA) neural network for rumor embedding and classification. They first modeled the propagation structure of each rumor as a set of propagation paths where each path represents the source post in a different context. Then they aggregated all paths to obtain the whole propagation structure. In addition, they utilized a neural topic model in the Wasserstein autoencoder (WAE) framework to capture insensitive stance patterns. Wang et al. [28] observed that the various posts of each rumor event will debate its realness over time. Different individuals have different emotional reactions to event. They firstly employed an automatic constructive method to develope a Sentiment Dictionary (SD) to capture human emotional reactions to different events. Then they elaborated a Two-step Dynamic Time Series (TsDTS) algorithm to retain the time-span distribution information of events. Finally, they proposed a two-layer Cascaded Gated Recurrent Unit (CGRU) model based on the SD and TsDTS. Shu et al. [29] found that social context in the process of news spreading on social media has formed inherent relationships among the publisher, the news and the users. They proposed a framework of modeling publisher-news and user-news interaction relations to classify fake news.

Long Short Term Memory (LSTM), Convolutional Neural Networks (CNN) and RNN are used frequently. Yuan et al. [30] thought that rumor identification models based on deep neural networks can be further improved by modifying the underlying architecture. Thus they proposed a dilated-block-based convolutional network (DBCN). It stacks dilated blocks to achieve a wider receptive field and automatically extract features with less information loss. Tu et al. [31] proposed a CNN-based rumor detection framework with joint text and propagation structure representation learning. Ma et al. [32] proposed a method for rumor detection. It integrates entity recognition, sentence reconfiguration and ordinary differential equation network under a framework named ESODE. Asghar et al. [33] investigated rumor detection problem by exploring different deep learning models. They emphasized considering the contextual information in both forward and backward directions in a given text. The proposed system is based on LSTM with CNN, which effectively classify tweets into rumors and non-rumors. Chahat Raj et al. [34] built textual and visual modules to aid the research over fake news detection. They proposed a multi-modal Coupled ConvNet architecture. It fused the data modules and classified online news depending on its textual and visual content. Liu et al. [35] presented a CNN+RNN based time series classifier to detect fake news, its input is time-series in news forwarding paths.

### Graph neural network methods

Graph Neural Networks (GNN) is very efficient and popular for natural language processing in the past few years. Propagation patterns and structures of rumors can be well captured by GNN. Bian et al. [36] proposed a new bi-directional graph convolutional model to explore the propagation and dispersion of rumors through the top-down and bottom-up structures. Dang Dong et al. [37] presented a model which can locate several rumor sources in the case of an unknown propagation pattern. Aiming to solve rumor detection tasks under the framework of representation learning, Wu et al. [38] proposed a novel approach of constructing propagation graph through spreading structure of posts on Twitter, and they applied an algorithm of gated graph neural networks to generate powerful representations for nodes in forwarding graph. Chen et al. [39] proposed a diffusion-based rumor detection model to explore the full-scale diffusion patterns of information. It utilizes graph neural networks to learn the macroscopic diffusion of rumor propagation, and uses bidirectional recurrent neural networks to capture microscopic diffusion patterns while considering user-time series.

Some research takes both spreading features, semantic information and content features. Lu et al. [40] exploited graph-aware co-attention networks based on source posts and series of no-comment retweet users. It could highlight suspicious retweeters and words to predict whether the source is a rumor. Yu et al. [41] created a GCN-based model that took both static characteristics like user information, text content and dynamic features such as rumor diffusion. Serveh Lotfi et al. [42] proposed a model using GCN to detect rumor conversations. The reply trees and the user graphs were extracted from conversations. The structural information of graphs, the contents of conversation tweets and the information of how users interacted in the conversation were obtained by modeling the reply trees and the user graphs respectively. Then the three modules were combined to detect rumors. Zhong et al. [43] found that there were not efficient methods to integrate the semantic information from content-related posts, to preserve the structural information for the reply relationship, and to properly handle posts that dissimilar to those in the training set. Thus, they proposed TopicPost-Comment Graph Neural Network (TPC-GCN) to overcome the first two limitations and extended the model to Disentangled TPC-GCN (DTPC-GCN). TPC-GCN integrates the graph structure and content of topics, posts and comments for controversy detection on the post level. DTPC-GCN disentangles topic-related and topic-unrelated features and dynamically fuse. Chen et al. [44] discovered that there was a problem of imbalanced data in rumor detection. They proposed a knowledge graph-based rumor data augmentation method, named Graph Embedding-based Rumor Data Augmentation (GERDA), to simulate the generation process of rumor from the perspective of knowledge.

Our proposed model is inspired by the GNN. Since GNN can learn the high-level representations of words in each document, and GCN can capture the structure of rumor propagation, we consider adopting the rumor detecting model with dual-grained hierarchical structured GNNs. In our model, the event graphs are constructed to represent rumor propagation. GCN updates text representations in event graphs. The text graphs are built to represent co-occurrence relations among words. GNN generates fused word-text vectors and further updates them to form final representations by constructing co-occurrence graphs of words. We optimize our model components to improve the accuracy of the method.

## Preliminaries

We introduce some essential concepts that are necessary for the proposed method. The notation in the paper is illustrated as follows.

In order to take full advantage of retweet post features and rumor propagation structures, event is used as the basic unit in our research. First, we define a rumor detection dataset as a set of events *C* = {*c*_1_, *c*_2_,..., *c*_*n*_}, where *c*_*i*_ is the *i*-th event and *n* is the number of events. ${c_{i}}=\{{{c_{0}^{i}}}, ~{{c_{1}^{i}}},...,~ {{c_{m}^{i}}}\}$, where ${{c_{0}^{i}}}$ is the source tweet and ${{c_{j}^{i}}}$ is the *j*-th responsive post of ${{c_{0}^{i}}}$. Graph *G*_*i*_ = (*N*_*i*_, *E*_*i*_) denotes the propagation graph of event *c*_*i*_, where node set ${N_{i}}=\{{{c_{0}^{i}}}, ~{{c_{1}^{i}}}, ...,~ {{c_{m}^{i}}}\}$ and ${E_{i}}=\{{e_{pq}^{i}} \vert p,q=0,1,...,m\}$ represents the set of edges from responded tweet to the retweet post. Number *m* denotes the number of retweet posts in event *c*_*i*_. For example, if event ${c_{1}^{i}}$ is a response to event ${c_{0}^{i}}$, there will be an edge between event ${c_{0}^{i}}$ and ${c_{1}^{i}}$, i.e., $e_{01}^{i}$. Matrix ${A_{i}}\in \{0,~1\}^{{(m+1)}\times {(m+1)}}$ denotes the adjacency matrix of event *c*_*i*_ where

1 $$ a_{pq}^{i}=a_{qp}^{i}=\left\{ \begin{array}{cl} 1 & ,~if ~e_{pq}^{i} ~exists\\ 0 & ,~otherwise. \end{array}\right. $$

Matrix $X_{i}=[{x_{0}^{i}},~{x_{1}^{i}},...,~{x_{m}^{i}}]^{T}$ denotes the text feature matrix extracted from the posts in *c*_*i*_, where ${x_{0}^{i}}$ represents the text feature vector of ${c_{0}^{i}}$ and ${x_{j}^{i}}$ represents the text feature vector of ${c_{j}^{i}}$. The algorithm of constructing event graphs is shown as the Algorithm 1. 
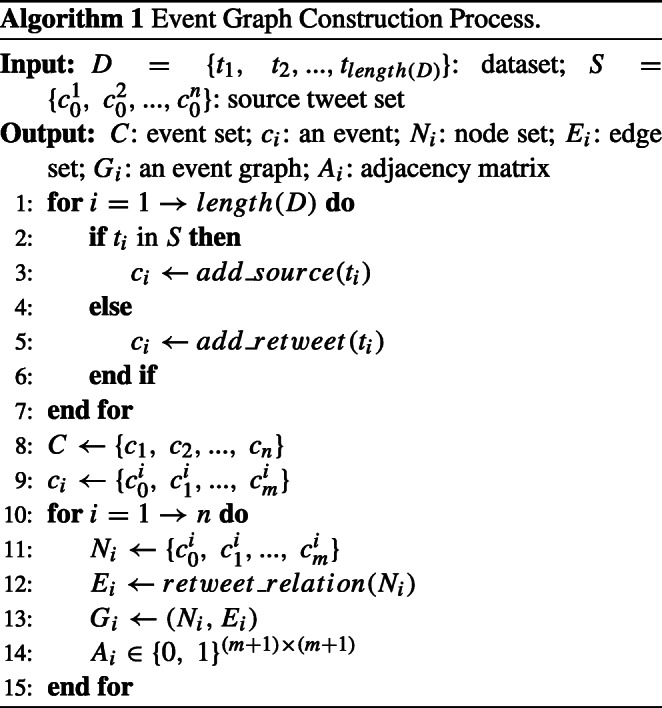


Then we extract source tweets $S=\{{c_{0}^{1}},~{c_{0}^{2}},...,{c_{0}^{n}}\}$ in the rumor detection dataset. Define a textual document graph $G^{\prime }_{i}=(Node_{i},~Edge_{i})$ for source tweet ${c_{0}^{i}}$, where $Node_{i}=\{nod{e_{1}^{i}},~nod{e_{2}^{i}},...,~nod{e_{r}^{i}}\}$ and $nod{e_{j}^{i}}$ is a word in the text of source tweet ${c_{0}^{i}}$. The parameter *r* denotes the number of words in document ${c_{0}^{i}}$. $Edge_{i}=\{edge_{st}^{i}\vert s,t=0,...,r\}$ represents the co-occurrence relation between words which describes the relationship of words that appear in the same sliding window. For example, if there is a co-occurrence relationship between nodes $nod{e_{1}^{i}}$ and $nod{e_{2}^{i}}$, there will be an edge between them, i.e., $edge_{12}^{i}$. Denote $A^{\prime }_{i}\in \{0,~1\}^{r\times r}$ as the adjacency matrix of textual document ${c_{0}^{i}}$ where

2 $$ a_{st}^{'i}=a_{ts}^{'i}=\left\{ \begin{array}{cl} 1 & ,~if ~edge_{st}^{i} ~exists\\ 0 & ,~otherwise. \end{array}\right. $$

Matrix $F_{i}=[{f_{1}^{i}},~{f_{2}^{i}},...,~{f_{p}^{i}}]^{T}$ denotes the word feature matrix extracted from the words in source tweet ${c_{0}^{i}}$, where vector ${f_{j}^{i}}$ represents a word feature vector of word $nod{e_{j}^{i}}$. The algorithm of constructing text graph is shown as Algorithm 2. 
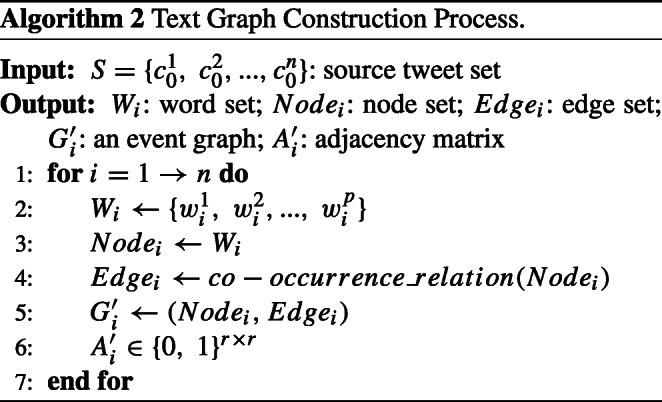


In addition, each event *c*_*i*_ is related to a label *y*_*i*_ ∈{*F*, *T*} (i.e., False Rumor or True Rumor). Given the dataset, we describe this task as a supervised classification problem that learns a classifier $f:C\rightarrow Y$ to predict the label of an event based on textual content and propagation structure, where set *C* are the event sets and set *Y* are the sets of labels.

## HAGNN rumor detection model

In this section, we propose a GNN-based bi-level feature aggregation method for rumor detection, named as *Hierarchically Aggregated Graph Neural Networks* (HAGNN). The core idea of HAGNN is to learn both word and text granularity high-level representations from text content and event propagation to detect rumors.

**Fig. 1:**
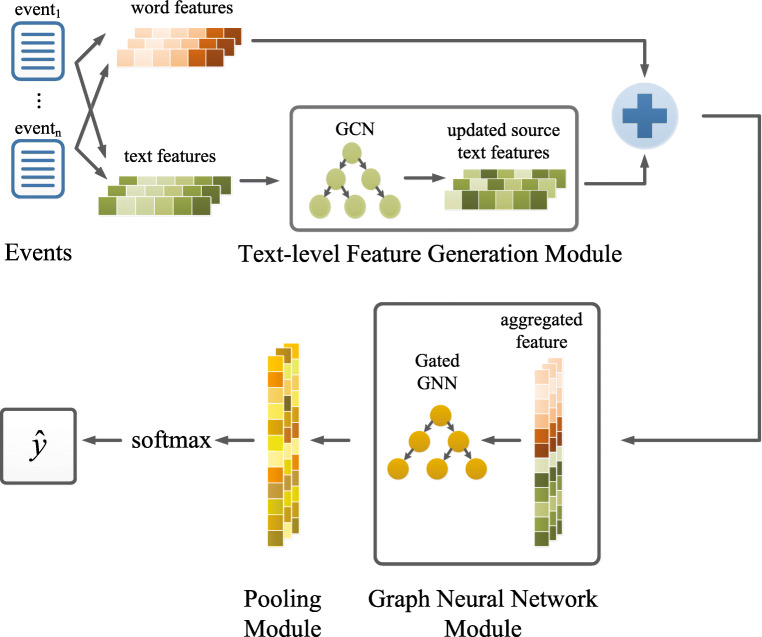
Our HAGNN rumor detection model

The rumor detection model consists of three modules, which are the text-level feature generation module, the graph neural network module and the pooling module, as shown in Fig. 1. Specifically, the text-level feature generation module captures textual content features from both source tweets and retweet posts. GCN is employed to obtain updated representations of text contents with event propagation structure. The graph neural network module uses Gated GNN to update word-text aggregated representations. We design the pooling module to aggregate node vectors and get the final representation vector of the entire graph.

### Text-level feature generation module

As is known to all, it is necessary to make the best of the retweet post information and propagation relationship. Therefore, we propose the text-level feature generation module. Adding propagation structure and retweet features, the source tweet features are enhanced and the performance of detecting rumors is improved. Based on the retweet relationships, we construct an event graph *G*_*i*_ = (*N*_*i*_, *E*_*i*_) for event *c*_*i*_. Then let matrix $A_{i}\in \mathbb {R}^{m_{i}\times m_{i}}$ be its corresponding adjacency matrix of *c*_*i*_. Matrix *A*_*i*_ contains the edges from responded tweets to the retweet posts.

After constructing the event graph *G*_*i*_, GCN generates text updated representation of event *c*_*i*_ based on matrices *A*_*i*_ and *X*_*i*_. The propagation function on the (l + 1)-th layer in GCN is as follows:

3 $$ {H^{(l+1)}=\sigma(\tilde{D}^{-\frac{1}{2}} \tilde{A} \tilde{D}^{-\frac{1}{2}} H^{(l)} W^{(l)}),~} $$

where *H* is the feature of the layer, $\tilde {A}=A+I$ is the adjacency matrix with added self-connections, $\tilde {D}_{ii}={\sum }_{j} \tilde {A}_{ij}$ is the degree matrix of $\tilde {A}$, and *W* represents the layer-specific trainable weight matrix. *σ*(⋅) denotes an activation function, for instance, the function *ReLU*(⋅) = *max*(0,⋅). The normalized symmetric adjacency matrix $\hat {A}=\tilde {D}^{-\frac {1}{2}} \tilde {A} \tilde {D}^{-\frac {1}{2}}$.

Then the event graph *G*_*i*_ is imported into GCN. The forward propagation function Z is as follows:

4 $$ {Z=f(X,A) = softmax(\hat{A} \cdot ReLU(\hat{A} \cdot ReLU(\hat{A} XW^{(0)})W^{(1)})W^{(2)}).~} $$

Here, weight $W^{(0)}\in \mathbb {R}^{C\times H}$ is an input-to-hidden weight matrix for the first hidden layer with *H* feature maps. Weight *W*^(1)^ is a hidden weight matrix, while weight *W*^(2)^ is a hidden-to-output weight matrix.

The GCN model is treated as a pre-trained model that saves parameters after stopping training. Then the saved parameters and a modified GCN model is used to generate the source tweet updated representation of event *c*_*i*_. Dropout technology is applied to avoid over-fitting.

### Graph neural network module

Using only source post text features for rumor detection will probably lead to one-sided results. Therefore, we consider hierarchical aggregation. The word features are aggregated with the updated text features produced in the text-level feature generation module. Base on the textual document graph $g_{i}^{\prime }=(Node_{i},~Edge_{i})$ of event *c*_*i*_, GRU is used on graph $g_{i}^{\prime }$ to learn the embeddings of word nodes. Specifically, here the word node refer to hierarchically aggregated word-text features. The nodes receive information from their neighbors, then selectively decide which to be saved and which to be got rid of, finally, merge the stayed information with their representations to update. The formulas of the operations are:

5 $$ a^{t}=Ah^{t-1}W_{a}, $$

6 $$ z^{t}=\sigma (W_{z}a^{t}+U_{z}h^{t-1}+b_{z}), $$

7 $$ r^{t}=\sigma (W_{r}a^{t}+U_{r}h^{t-1}+b_{r}), $$

8 $$ \tilde{h}^{t}=tanh(W_{h}a^{t}+U_{h}(r^{t}\odot h^{t-1}+b_{h})), $$

9 $$ h^{t}=\tilde{h}\odot z^{t}+h^{t-1} \odot (1-z^{t}),~ $$

where matrix $A\in \mathbb {R}^{\vert \mathcal {V} \vert \times \vert \mathcal {V} \vert }$ is the adjacency matrix, function *σ* is the sigmoid function, *z*^*t*^ is the update gate while *r*^*t*^ is the reset gate. *W*,*U* and *b* are trainable parameters.

By using GRU, we get the updated word-text aggregated features. Then the representations are further updated. The equation is given as follows:

10 $$ h_{v}=\sigma (f_{1}({h_{v}^{t}})) \odot tanh(f_{2}({h_{v}^{t}})),~ $$

where ${h_{v}^{t}}$ is the node representation that GRU generates. Functions *f*_1_ and *f*_2_ are multilayer perceptrons (MLP) where the former one is an attention weight and the latter one is a non-linear feature transformation.

### Pooling module

The target of our task is to classify the graph structure after N iterations of the neural network. Accordingly, we need to aggregate the node vectors into a vector. Considering that each word plays a certain role in the text, we average the word nodes that denote the word-text features. In addition, the role of keywords should be more explicit, thus we employ the maximum pooling, as shown in (11):

11 $$  {h_{G}=\frac{1}{\vert \mathcal{V} \vert} \sum\limits_{v \in \mathcal{V}} h_{v}+max(h_{1},\dots ,h_{u}).~} $$

The maximum pooling is selecting the largest value of all nodes in the same dimension as the final output of each dimension. Here, *h*_*G*_ is the graph representation, set $\mathcal {V}$ is the node set of a graph and vector *h*_*j*_ is the ultimate updated word representation of each node.

Then the predicted label of event *c*_*i*_ is calculated by using a softmax layer after obtaining the graph-level vector *h*_*G*_:

12 $$ {\hat{y}_{G}=softmax(W \cdot h_{G}+b),~} $$

where *W* and *b* are weights and bias respectively, $\hat {y}_{G} \in \mathbb {R}^{1\times K}$ is a probability vector for all the classes used to predict the label of event *c*_*i*_, and *K* is the number of categories.

The algorithm of our model is shown as Algorithm 3. 
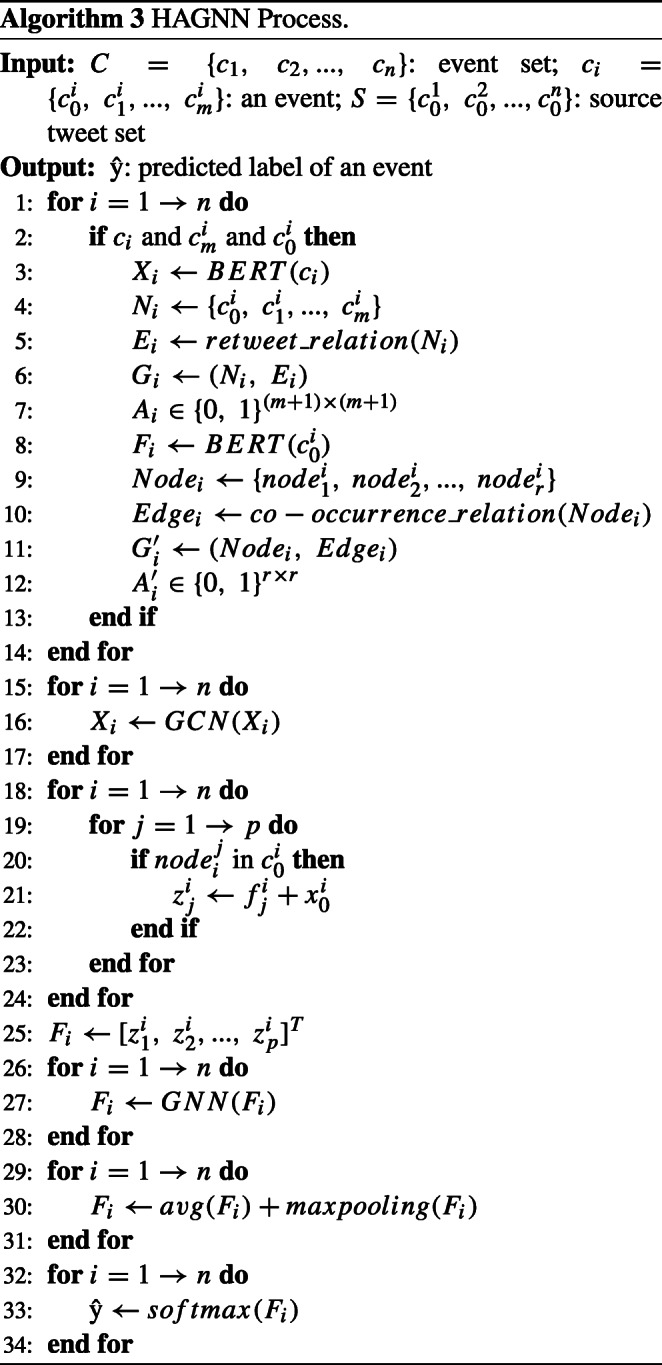


## Experiments and analysis

### Dataset

We choose the dataset in Sina Weibo named Weibo to assess our proposed method. It includes two categories of labels, which are False Rumor (F) and True Rumor (T). In the dataset, nodes in the event propagation graph refer to tweet posts while nodes in the source tweet-text graph refer to words in text content. Edges in the event graph represent the forwarding relationship, and edges in the source text graph represent the co-occurrence relationship. Besides, the other dataset in Sina Weibo called CED is also used to assess the model. It has the same structure as the Weibo dataset. The statistics of the Weibo dataset and the CED dataset are shown in Table 1.

**Table 1:** Statistics of the datasets

Statistic	Weibo	CED
# of events	4664	3387
# of Rumors	2351	1538
# of Non-rumors	2313	1849
# of Posts	3,805,656	1,217,212
# of Users	2,746,818	771,960

### Experiment settings

The pre-trained BERT model [45] with 12 layers and 256 dimensions is utilized to extract vectors. In the text-level feature generation module, it is used to extract text features for the source tweets and the first 15 retweets of each event. In the graph neural network module, it is applied to extract word vectors in every source tweet. The output of the penultimate layer in BERT is taken as the original text and word representations.

The proposed model uses GCN in the text-level feature generation module. The unit number in the input layer and the hidden layer of GCN are 16 and 128, while the unit number in the output layer is 32. In the graph neural network module, 256-dimension word feature vectors are concatenated with 32-dimension text representations. The Gated GNN is employed to update the word-text aggregated representations. The size of the sliding windows in the module is 5 words, which means there is a co-occurrence relation among 5 words in the window at the same time. The unit number in the hidden layer of GGNN is 96. Gradient descent is used to update parameters. Moreover, in both GCN and GGNN, the Adam algorithm is applied to optimize the model, the dropout rate is 0.5. In the graph pooling module, a maximum pooling algorithm and an average operation are utilized to update features.

### Typical methods

We compare the proposed model with the following typical methods, including:

- SVM-RBF [46]: a rumor detecting method using manual features and SVM classifier with RBF kernel function. The handworked features are extracted from Sina Weibo.
- RvNN [10]: a rumor detecting approach based on tree-structured recursive neural networks. It learns tweet representations via event propagation.
- TextING [47]: a text classification model with Gated GNN and MLP. In the graph, nodes represent words and edges represent word co-occurrence relations.
- TextGCN [13]: a GCN model applied in text classification field. It uses word co-occurrence and document word relations to build a text graph. It utilizes the one-hot vector as the word features and apply TF-IDF as the edge weight in the text graph.
- PPC_RNN+CNN [35]: an early detection approach of fake news through classifying propagation paths. It constructs the paths as multivariate time series and build a time series classifier incorporating RNN and CNN.

### Evaluation metrics

To evaluate the classification model, a confusion matrix between the prediction results and the real label is generated. The confusion matrix is shown in Table 2. TP (True Positive) value represents the number of positive samples predicted to be positive; FP (False Positive) value indicates the number of negative samples predicted to be positive; FN (False Negative) value represents the number of positive samples predicted to be negative; and TN (True Negative) value indicates the number of negative samples predicted to be negative.

**Table 2:** Confusion Matrix

		Actual	
		Positive	Negative
Predicted	Positive	TP	FP
	Negative	FN	TN

We use the four indicators to evaluate the model: Accuracy, Precision, Recall and F1-score. They can be calculated by the following (13), (14), (15) and (16):

13 $$  Accuracy=\frac{TP+TN}{TP+TN+FP+FN} $$

14 $$  Precision=\frac{TP}{TP+FP} $$

15 $$  Recall=\frac{TP}{TP+FN} $$

16 $$  F1=\frac{2 \times Precision \times Recall}{Precision+Recall} $$

Accuracy indicates the proportion of correctly classified results in the total results. Precision represents the share of the correctly predicted results among all results predicted to be positive. Recall indicates the proportion of correctly predicted results in all real-positive results. F1-score represents the comprehensive results of Precision and Recall. It ranges from 0 to 1, 0 means the worst performance while 1 means the best.

### Result analysis

**Fig. 2:**
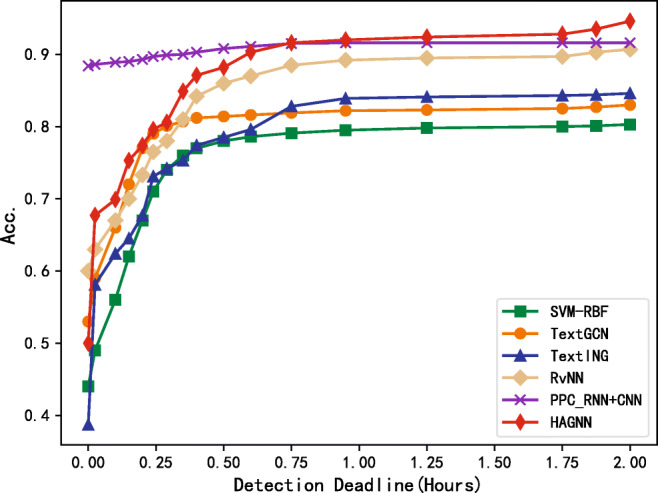
Comparison of different methods on Weibo dataset

As shown in Fig. 2, deep learning approaches perform better than those using manually extracted features. The detection speed of HAGNN increases rapidly in the beginning. It can be inferred that the high-level aggregated representations are helpful for model learning. Our proposed model HAGNN yields better than the referenced methods.

Results of baseline models and the proposed model are shown in Tables 3 and 4. The symbol “*” denotes values that are taken from the original papers. In terms of Accuracy, the proposed model improves TextGCN, TextING, RvNN, and PPC_RNN+CNN by 12 percentage points, 11.5 percentage points, 4.9 percentage points, and 4.1 percentage points respectively on the Weibo dataset. On the CED dataset, the proposed model improves TextING, TextGCN by 4 percentage points, 0.9 percentage points. This is because we employ both GCN and GNN structures. However, the combination of a complex text classification approach and large vectorization models may not be helpful for accuracy. In addition, models with propagation features have higher scores in the evaluation. It is vital of employing different types of features to enhance text vectors for helping to classify the event.

**Table 3:** Results on the Weibo dataset (F:False Rumor,T:True Rumor)

Method	Class	Accuracy	Precision	Recall	F1
SVM-RBF^∗^	F	0.818	0.822	0.812	0.817
	T		0.815	0.824	0.819
TextGCN	F	0.837	0.809	0.840	0.824
	T		0.862	0.835	0.848
TextING	F	0.842	0.851	0.844	0.848
	T		0.832	0.839	0.836
RvNN^∗^	F	0.908	0.912	0.897	0.905
	T		0.904	0.918	0.911
PPC_RNN+CNN^∗^	F	0.916	0.884	0.957	0.919
	T		0.955	0.876	0.913
HAGNN	F	**0.957**	**0.949**	**0.983**	**0.966**
	T		**0.970**	**0.917**	**0.943**

The bold entries are used to highlight the results of our proposed model

**Table 4:** Results on the CED dataset (F:False Rumor,T:True Rumor)

Method	Class	Accuracy	Precision	Recall	F1
TextING	F	0.842	0.851	0.844	0.848
	T		0.832	0.839	0.836
TextGCN	F	0.873	0.913	0.846	0.878
	T		0.833	0.906	0.868
**HAGNN**	F	**0.882**	**0.932**	**0.873**	**0.902**
	T		**0.810**	**0.895**	**0.850**

The bold entries are used to highlight the results of our proposed model

Next, some comparison experiments is performed to compare the influence of different factors in HAGNN. We conducted repeated experiments under the same condition and took the average of the experiments as the ultimate result of each experimental group.

**Fig. 3:**
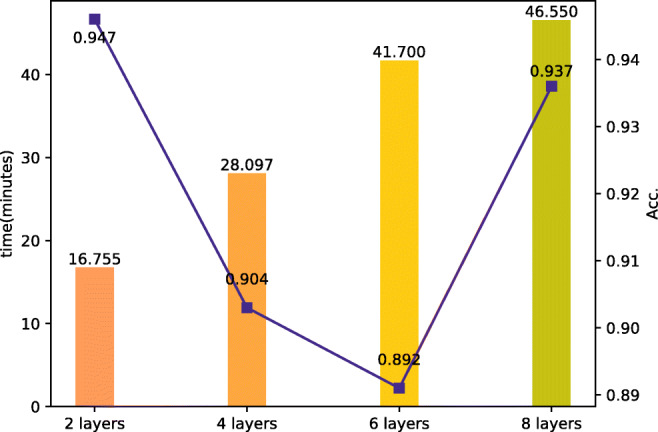
Results on the comparison of GNN layer number

**Fig. 4:**
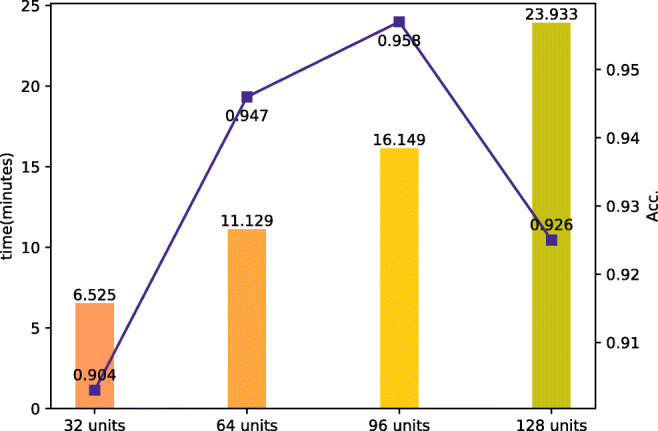
Results on the comparison of GNN unit number

**Fig. 5:**
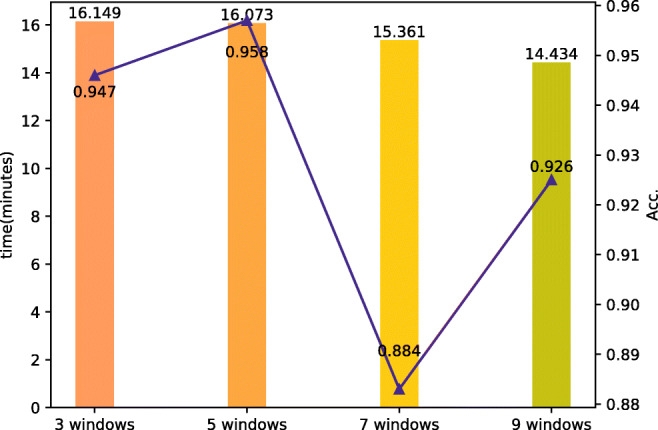
Results on the comparison of GNN window size

1. The effect of the GNN layer number: We discussed the impact of the GNN layer number on the time and accuracy of the model. The experiments are compared among four parts, which are the 2-layer group, the 4-layer group, the 6-layer group and the 8-layer group. Figure 3 shows the influence of the GNN layer number on time and accuracy. The detecting time prolongs as the number of layers increases. Although the accuracy rebounds in the 8-layer group, the overall trend of the accuracy is decreasing. The 2-layer set has the best accuracy of 0.947. It can be concluded that it is unnecessary for the GNN-structured neural network to have deeper architecture, the number of the GNN layer that can get excellent performance is 2 to 3.
2. The effect of the GNN unit number: We analyze the influence of the unit number in a GNN layer on both time and accuracy of the proposed model. The experiments are compared among four sets, which are the 32-unit set, the 64-unit set, the 96-unit set and the 128-unit set. Figure 4 shows the effect of the GNN unit number on time and accuracy. It takes more time to complete detecting with the GNN units increase. In addition, the accuracy reaches its peak when GNN has 96 units. It can be deduced that the time spent on detecting is positively correlated with the number of units in GNN. But the accuracy is not positively correlated with the number of units in GNN. The best accuracy will be more likely on the GNN with 96 units in a layer than that on the GNN with 128 units.
3. The effect of the GNN window size: We also study the impact of the GNN sliding window which works at the stage of word co-occurrence graph construction in the graph neural network module. The window is used for calculating which words have a co-occurrence relationship. It keeps sliding when the words in it have built the relationship of co-occurrence. It will not stop until it reaches the end of an event text. The experiments are compared among four different sliding windows, which are the 3-window set, the 5-window set, the 7-window set, and the 9-window set. Figure 5 shows the influence of the GNN window size on time and accuracy. The time spent on detecting is negatively correlated with the size of windows in GNN. The accuracy is the best at the 5-window set with 0.958, but declines when the sliding window size is 7, then rebounds when the window size is 9.

### Ablation study

To analyze the effect of each part in HAGNN, a set of exploratory experiments is conducted to study the relative contribution of each component in our model.

**Fig. 6:**
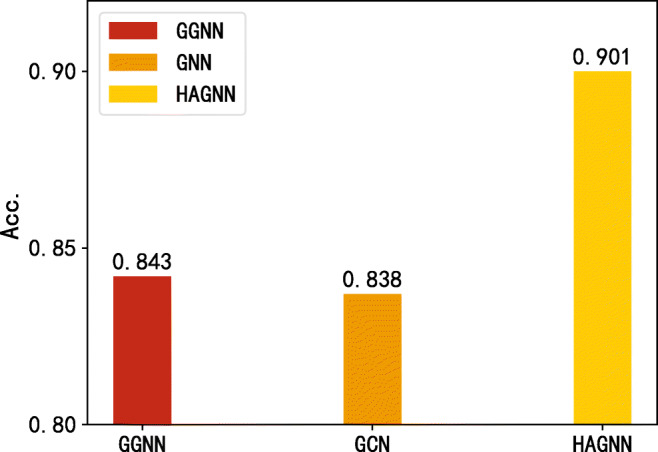
The comparison of our model, GGNN and GCN

**Fig. 7:**
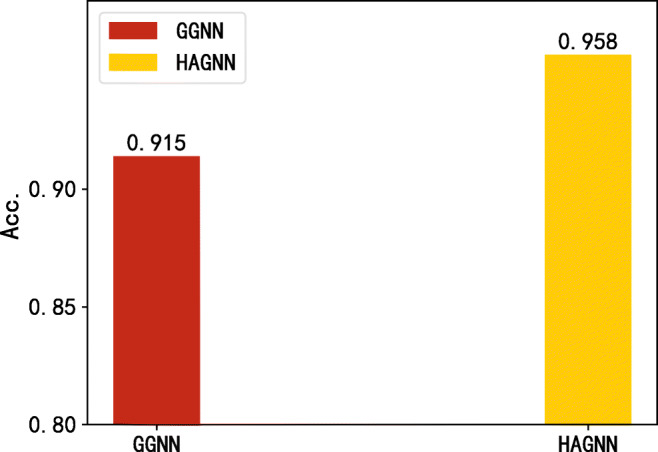
The comparison of our model and GGNN

Gated GNN and GCN are used to illustrate the role they play in the HAGNN. Figure 6 shows that our proposed model outperforms the GGNN and GCN by 5.8 percentage points and 6.3 percentage points respectively in the case of using only word features. It can be inferred that GGNN and GCN are indivisible in our model, either of them plays a significant part in HAGNN. Figure 7 shows that the HAGNN model performs better than GGNN in the circumstance of using both source-retweet text-granularity features and word-granularity features. The input of GGNN is original word-text features without GCN-updating, while the input of HAGNN is the GCN-updated word-text features. We can conclude that word-text aggregated features with propagation structures and GCN are conducive for model performance improvement.

## Conclusions

In this paper, we proposed an improved GNN-based model named HAGNN for rumor detection on Sina Weibo. GCN was utilized to generate source text granularity features by constructing events as graphs. The event graphs helped to update source text features by retweet post features. The text-granularity features were aggregated with word-granularity features to make hierarchically fused features, which denote word nodes in source text graphs. Besides, we adopted GNN with source text graphs to update, generate the ultimate representations of events and then predict. The experimental results on the Sina Weibo dataset demonstrated that the GNN-based method outperformed baselines in terms of both efficiency and accuracy. In particular, the HAGNN model achieved performance by considering different hierarchies of both word features and source-retweet features with propagation structure.

It is an important direction for public opinion monitoring to solve the problem on how to adapt the rumor detecting model to a changing online social environment and maintain a relatively balanced accuracy. In the future, we will further explore: 1) bias, discrimination and deception in social media. 2) environmental, user factors and phrasing characteristics in rumor propagation. 3) fake news detection in a dynamic media public environment. 4) word and emoticon affective expression tendency.

## Declarations

This work was supported in part by the National Natural Science Foundation of China (Grant No. U1703261).
